# Structural Optimization of Cannabidiol as Multifunctional Cosmetic Raw Materials

**DOI:** 10.3390/antiox12020314

**Published:** 2023-01-29

**Authors:** Xuelian Chen, Jie Su, Runan Wang, Rui Hao, Chenggong Fu, Jingjing Chen, Jiazhong Li, Xin Wang

**Affiliations:** School of Pharmacy, Lanzhou University, 199 West Donggang Rd., Lanzhou 730000, China

**Keywords:** cannabidiol, virtual screening, anti-oxidation, anti-inflammation, anti-wrinkle, whitening

## Abstract

Cannabidiol (CBD), derived from the plant cannabis, can be used in the cosmetics industry for its antioxidant, anti-inflammatory, anti-wrinkle and whitening effects. However, CBD is purified from the hemp plant extract, its source is very limited and under strict control. So in this study, computational and experimental methods were combined to search for novel CBD substitutes with high biology potencies. The action mode between CBD and target protein cannabidiol receptor 1 was studied to find the key skeleton, which was used to virtually screen a natural products database to search for compounds with 70% similarity. The hit compounds with high docking scores were selected for the ABTS and DPPH free radical scavenging experiments for antioxidant evaluation. The effects on the expressions of nitric oxide (NO), interleukin-6 (IL-6), COX-2 and iNOS in RAW264.7 cell line were detected to demonstrate their anti-inflammatory abilities. The effect of anti-wrinkle ability were evaluated by detecting the extracellular matrix, such as collagen, elastin, fibronectin and reactive oxygen species (ROS) in HFF-1. The effects on melanin production and tyrosinase activity in Bb16F10 were also detected. As a result, two compounds were found to be superior to cannabidiol, in terms of antioxidant, anti-wrinkle and whitening efficacy with a lower cytotoxicity.

## 1. Introduction

The appearance of skin is a major factor used to roughly estimate a person’s facial attractiveness [1]. When affected by exogenous factors (ultraviolet, dust particles, drugs, etc.) [2,3,4] or endogenous factors (ischemia, metabolic processes, hormones, etc.) [5], skin may be infected with some pathogens. In response to these infections, the body will produce oxidative stress, which induces the body’s immune system [6]. Macrophages start to work by activating nuclear factor kappa-B (NF-κB) channels. Overprotection of the immune system is responsible for the development of skin problem, such as dermatitis, winkling and skin dryness [1]. Oxidative stress can also lead to the formation of reactive oxygen species (ROS) in the skin, which result in the melanin biosynthesis and DNA damage [7,8,9,10]. Furthermore, ultraviolet (UV) irradiation can also promote the production of a large amount of ROS in the dermis and epidermis, because UV is a high-energy light wave, which can promote the oxidative stress reaction of some skin biomolecules containing chromophores. UV irradiation appears to be a key factor in skin aging and fibroblast apoptosis.

The reactive oxygen species (ROS) are essential to life, as they are involved in several biological functions. However, when produced at high levels, ROS become highly harmful, leading to the cellular damage. ROS can promote the expression of activator protein 1 (AP-1) and its upstream signal transduction proteins, including mitogen-activated protein kinases (MAPK) [11]. The MAPKs family, including p38 MAPK, c-jun amino terminal kinase (JNK) and extracellular signal regulated kinase (ERK) [12], induces the expression of matrix metalloproteinases (MMPs). MMPs further catalyze the decomposition of extracellular matrix (ECM) [2,5,13,14], such as collagen, elastin and fibronectin [5,15], which widely exist in the dermis [16], maintaining skin firmness, strength, smoothness and elasticity [2]. In addition, ROS can lead to the collagen degradation and reduce its production by inhibiting the proliferation of fibroblasts. Interaction of ROS with biomacromolecules, such as DNA, lipids and proteins [17], can accelerate the senescence of the fibroblasts, which in turn generates more ROS. It is a vicious cycle [18]. Furthermore, the occurrence of inflammation can also promote the generation of MMPs [5,19], leading to the degradation of ECM [20].

Additionally, ultraviolet light induces keratinocytes in the epidermis to secrete α-melanocyte-stimulating hormone (α-MSH). α-MSH binds to melanocortin-1 receptor (MC1R) of melanocytes activating microphthalmia transcription factor (MITF) via the MAPK [21] or cAMP-PKA-CREB signaling pathway [7,11,22,23]. MITF can modulate melanocyte differentiation and melanogenesis. Furthermore, MITF can regulate the expression of tyrosinase and other melanozymes. Tyrosinase, which is considered as a rate-limiting enzyme, catalyzes the first two steps of melanogenesis, hydroxylates tyrosine to 3,4-dihydroxyphenylalanine (DOPA) [24] and the further oxidation of DOPA into DOPA quinone [15,24]. Tyrosinase-related protein (TRP-1 and TRP-2) resides in melanosome can also regulate melanin generation [25,26]. Following a couple of reactions, L-dopaquinone converts into 5,6-dihyroxyindole, which eventually becomes melanin, catalyzed also by tyrosinase [21]. This process is a signal cascade reaction of α-MSH-MITF [11,24].

It is reported that cannabidiol (CBD), derived from the plant cannabis, can enhance the activities of antioxidant enzymes, such as glutathione peroxidase (GSH-Px) and superoxide dismutase (SOD) [27,28,29,30], thereby reducing the content of reactive oxygen species. In 2019, a study showed that CBD has whitening and brightening effects [31]. Actually, CBD has many biological characteristics, such as regulating immune response and treating depression, excellent anti-inflammatory effect by inhibiting the generation of TNF-α, IL-1β, nitric oxide (NO), iNOS and COX-2 [28,30,32,33]. CBD can also inhibit the activity of MMPs, which further prevent the ECM from being degraded, thus maintaining the level of collagen, elastin and fibronectin. However, the main source of CBD is purified from hemp plant extracts, which results in its strict supervision by the government. Hemp seed fruit, hemp seed oil, hemp leaf extract and CBD are listed as banned ingredients in cosmetics in many countries.

So, this study aims to find CBD substitutes owning similar or even better anti-inflammatory, antioxidant, anti-wrinkle and whitening effects that can be used in the cosmetics. At first, the action mode between CBD and the target protein canadiol receptor 1 (CB1) [20] was investigated to find the key skeleton related to its biological characteristics. The key skeleton was placed into a natural products database to search for compounds with a 70% structural similarity. Then molecular docking was used to fish out compounds with similar binding modes with CBD. As the anti-wrinkle, anti-inflammatory and whitening abilities of the compounds are closely related to the antioxidant ability, it is necessary to test the antioxidant capacity. The antioxidant capacity of the hit compounds was tested by DPPH, ABTS radical scavenging experiments and fluorescent stained flow cytometry, The MTT assay, western blotting and ELISA, etc., are also used to evaluate the skin care efficacy of these compounds.

## 2. Materials and Methods

### 2.1. Materials and Chemicals

Complete medium for RAW264.7 was bought from Procell (Procell, Wuhan, China). Griess reagents were purchased from Nanjing Jiancheng Bioengineering Institute (Nanjing Jiancheng Bioengineering Institute, Nanjing, China). IL-6 kits were bought from Elabscience (Elabscience, Wuhan, China). iNOS antibody and COX-2 antibody were purchased from Signalway Antibody (Signalway Antibody, College Park, MA, USA) CBD and the hit compounds were purchased from TargetMol (TargetMol, Shanghai, China). Collagen kit, elastin kit and fibrin kit were bought from mlbio (mlbio, Shanghai, China). Apoptosis kit was bought from MultiSciences (MultiSciences, Zhejiang, China). Lipopolysaccharide (LPS) was purchased from Sigma-Aldrich (St. Louis, MO, USA). DPPH, ABTS, MTT and 2, 7-dichlorofuorescin diacetate (DCFH-DA) were bought from Solarbio Science & Technology (Solarbio, Beijing, China). α-MSH was bought from APExBIO (APExBIO, Houston, TX, USA). 

### 2.2. Computer-Aided Virtual Screening

**Figure 1:**
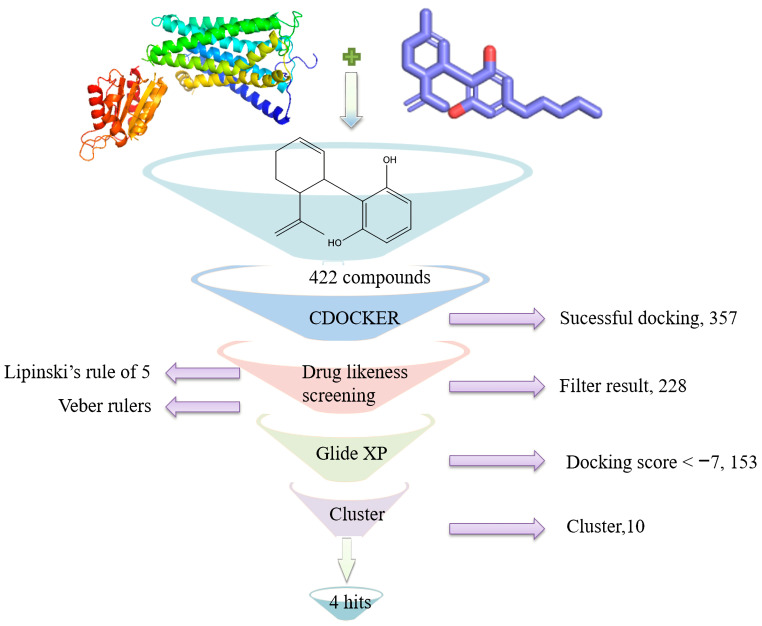
Virtual filtering process in this study.

Virtual screening is a common, efficient and economical method for drug design. The virtual screening process of this study is shown in Figure 1.

#### 2.2.1. CBD Substitutes Obtained and Primary Docking Screening

The cannabinoid receptor CB1, a member of the endocannabinoid system, is the target protein of CBD [34]. The CB1 receptor is involved in various physiological functions of the skin, such as inflammation, regulation of immune responses, proliferation and apoptosis [35]. The crystal structure of CB1 (PDB entry: 5TGZ) was obtained from the RCSB (PDB; https://www.rcsb.org/) (accessed on 17 October 2020) [36]. Accelrys Discovery Studio 2.5 software (DS 2.5) (Accelrys Software Inc., San Diego, CA, United States) was used to prepare the protein receptor. The location of antagonist AM6538 in the CB1 crystal structure was set as the active binding site. The structure of CBD was prepared using ligands tools. Then, the Glide XP (extra Precision) docking was implemented to explore the interaction between CBD and the target protein. Based on the docking results, the key CBD structural skeleton was determined. Compounds with 70% similarity to the key skeleton structure were searched in the Specs database and the InterBioScreen database CDOCKER protocol was used for the binding ability of the compounds to CB1. Compounds with better scores than CBD were retained for further analysis.

#### 2.2.2. Drug Likeness Evaluation

Lipinski’s rule of five and Veber’s rules were used to screen the drug likeness evaluation of the compounds. If a small molecule drug has less than ten hydrogen bond acceptors, five hydrogen bond donors, the calculated LogP (CLogP) <5 and a molecular weight (MWT) <500, it may show better pharmacokinetic properties. Veber’s rules refer to a compound with a rotatable bond ≤10 and a polar surface area of ≤140 Å^2^. If the compound meets the Veber’s rules, it has a higher drug likeness [26].

#### 2.2.3. High Precision Screening

We used Schrödinger’s Glide XP (extra precision) to dock these compounds with CB1. These compounds are given an OPLS_2005 force field. We set the pH to 7.0 + 2 to convert the compounds to the charged state. Schrödinger’s LigPrep module is used to generate different tautomers. Compounds with a docking score less than −7 were selected for further analysis [37].

#### 2.2.4. Similarity Searching

In order to obtain compounds with diverse structures, these compounds were clustered using hierarchical clustering in canvas module (Canvas, Schrödinger, LLC, New York, NY, USA). The cluster similarity threshold was set to 0.98. Selecting the candidate compounds required a combination of the following factors, receptor-ligand binding mode, docking score, molecular skeleton, *N*-heterocyclic compound, hydrocarbon group, amino and amide, halogen, etc. [38].

### 2.3. Antioxidant Capacity Evaluation

Following the virtual screening, the DPPH free radical scavenging assay and ABTS free radical scavenging assay were used to test their antioxidant effects.

#### 2.3.1. DPPH Radical Scavenging Activity

CBD and its derivative solution (0.015625–1.0 mg/mL) were prepared with distilled water. First, 40 μL each sample and 160 μL of 0.1 mg/mL ethanol solution of DPPH were mixed and reacted for 30 min in the dark [39]. The absorbance was measured at 520 nm [39]. The DPPH radical scavenging rate was calculated using the following formula: Y% = (A_0_ − A_1_)/A_0_ × 100, where A_0_ is the absorbance of the blank group (distilled water + DPPH), and A_1_ is the absorbance of the sample reaction (sample + DPPH).

#### 2.3.2. ABTS Radical Scavenging Activity

ABTS working solution was generated by mixing equal volumes of 2.45 mM potassium persulfate and 7 mM ABTS. The reaction was carried out in the dark for 12–16 h. Then, the radical solution obtained was diluted with PBS of pH 7.4 to an absorbance of 0.70 ± 0.05, measured at 405 nm. CBD and its derivatives solution (0.015625–1.0 mg/mL) were prepared with distilled water. First, 10 μL each sample and 200 μL ABTS working solution were mixed and reacted for 30 min in the dark. Then, the absorbance was measured at 405 nm [39]. The ABTS radical scavenging rate is calculated using the following formula: Y% = (A_0_ − A_1_)/A_0_ × 100, where A_0_ is the absorbance of the blank group (distilled water + ABTS working solution), and A_1_ is the absorbance of the sample reaction (sample +ABTS working solution).

### 2.4. Cytotoxicity Measurement of CBD and Its Derivatives

The RAW264.7 macrophage line, HFF-1 fibroblast and B16F10 melanoma cell were cultured with high glucose medium containing 13%, 15% and 10% fetal bovine serum (FBS), respectively, and 1% penicillin–streptomycin (P/S) was added to the medium at 37 °C in a 5% CO_2_-humidified air environment [40].

RAW264.7 cells were inoculated into 96-well plates at a density of 5 × 10^3^ cells per well. Then, 24 h later, the medium containing different concentrations of samples was added and cultured in a 37 °C cell culture box containing 5% carbon dioxide for another 24 h. Then, 15 μL MTT (5 mg/mL) was added to each well, and 150 μL triple solution was added after 4 h. We dissolved methylzan crystals overnight (the triad solution was made of 0.01 M hydrochloric acid solution, 5% isopropyl alcohol and 10% SDS). The absorbance at 570 nm was measured using an enzyme plate the next day [41]. The cell viability test procedure of B16F10 cells and HFF-1 cells were the same as RAW264.7 cell, except that they were cultured for three days after adding medium containing different concentrations of samples.

### 2.5. Anti-Inflammatory Activity Test

The Griess method was used to detect the changes in NO released by the cells in media containing different concentrations of compounds. The ELISA method was used to exam their effects on the release of IL-6, and the western blot was applied to analyze the expression level of COX-2 and iNOS.

#### 2.5.1. Measurement of NO and IL-6 Levels

RAW264.7 cells were inoculated into 96-well plates at a density of 2 × 10^4^ cells per well. Twenty-four hours later, medium containing different concentrations of compounds was added and cultured in a 37 °C cell culture box containing 5% carbon dioxide. Then, 2 h later, except for the control group, 1.5 μL LPS (final concentration: 1 μg/mL) was added and cultured for 24 h. Then, the supernatant was collected and treated with Griess reagent to detect NO levels. The absorbance of the supernatant at 520 nm was detected by a microplate reader. The expression of IL-6 was detected by ELISA method. The experiment was carried out according to the kit instructions.

#### 2.5.2. Western Blot Analysis

The cells were inoculated into 6-well plates at a density of 2 × 10^6^ cells per well and placed in an incubator for 24 h. Medium containing different concentrations of compounds was added. Two hours later, except for the control group, LPS (final concentration: 1 μg/mL) was added and cultured for 24 h. Cells were collected by centrifugal collection and the cell lysate was added. The mixture was placed on ice and shaken for 0.5 h, followed by centrifugation. A Bradford protein assay was used to determine the total protein concentrations; 10 μg protein from each sample was subjected to SDS-PAGE, transferred to BioBond nitrocellulose membranes, and then probed with the antibodies against iNOS and COX-2 (both in the ratio 1:2000). A horseradish peroxidase-conjugated goat anti-rabbit IgG antibody (1:5000) was used as the secondary antibody. Finally, enhanced chemiluminescence was used to visualize the antibody binding. The obtained images were analyzed by ImageJ software.

### 2.6. Antiwrinkle Activity Test

The MTT method was used to detect the repair effect of the compounds on HFF-1 cells damaged by UV radiation, and the ELISA method was used to detect the changes in the content of collagen, elastin and fibronectin in cells after adding CBD and its derivatives. The 2, 7-dichlorofuorescin diacetate (DCFH–DA, 10 μM) color method was used to detect the contents of fibroblasts’ intracellular reactive oxygen species. Annexin V-FITC/PI double staining cell apoptosis assay was used to detect the effect of compounds on the apoptosis of HFF-1.

#### 2.6.1. Repair Ultraviolet Radiation Damage Experiment

The cells were inoculated into 96-well plates at a density of 5 × 10^3^ cells per well and placed in an incubator for 24 h. Medium containing different concentrations of compounds was added. Fours hours later, except for the control group, UV intensity of 70 mW/cm was irradiated for 5 min. Following another 20 h, 15 μL MTT (5 mg/mL) was added to each well, and 150 μL triple solution was added after 4 h. Once the methylzan crystals were dissolved overnight (the triad solution was made of 0.01 M hydrochloric acid solution, 5% isopropyl alcohol and 10% SDS), the absorbance at 570 nm was measured using an enzyme plate, the next day.

#### 2.6.2. Detection of Collagen, Elastin and Fibronectin Levels

The cells were inoculated into 6-well plates at a density of 2 × 10^5^ cells per well and placed in an incubator for 24 h. Medium containing different concentrations of compounds was added. Four hours later, except for the control group, UV intensity of 70 mW/cm was irradiated for 5 min. Following 20 h of culture, HFF-1 cells were digested and cell precipitation was obtained by centrifugation. Collagen, elastin and fibronectin in cells were released by RIPA lysis solution, and the expression of collagen, elastin and fibronectin was detected by the ELISA method. The experiment was carried out, according to the kit instructions.

#### 2.6.3. Measurement of the ROS Content

The cells were inoculated into 6-well plates at a density of 2 × 10^5^ cells per well and placed in an incubator for 24 h. Medium containing different concentrations of compounds was added. Four hours later, except for the control group, UV intensity of 70 mW/cm was irradiated for 5 min. Following 20 h of culture, the cells were incubated with probes 2, 7-dichlorofuorescin diacetate (DCFH-DA, 10 µM) at 37 °C in 5% CO_2_-humidified air environment for 45 min. The excess probes were removed by PBS. Finally, the outcome was detected by flow cytometry.

#### 2.6.4. Detection the Effects on the Apoptosis of HFF-1 Cells

The cells were inoculated into 6-well plates at a density of 2 × 10^5^ cells per well and placed in an incubator for 24 h. Medium containing different concentrations of compounds was added. Four hours later, except for the control group, UV intensity of 70 mW/cm was irradiated for 5 min. Following 20 h of culture, HFF-1 cells were digested, cell precipitation was obtained by centrifugation. Then, after adding 10 μL PI staining solution, 5 μL Annexin V-FITC and 100 μL of working solution were added and left for 30 min at room temperature in the dark. The fluorescence of FITC and PI were measured using a flow cytometer and quantified using CytExpert software.

### 2.7. Determination of Melanin Content and Tyrosinase Activity

The cells were inoculated into 6-well plates at a density of 1 × 10^5^ cells per well and placed in an incubator for 24 h. Medium containing different concentrations of compounds was added. Four hours later, except for the control group, α-MSH was added and culture was continued for 48 h, then the medium was removed and washed three times with PBS. B16F10 melanin content was detected by the cleavage method. Following the addition of 800 μL 1 M NaOH solution containing 10% DMSO at 80 °C for 1 h, The absorbance at 405 nm was measured.

Tyrosinase activity was detected by the principle that tyrosine and its substrate will appear in color when combined; 720 μL 1% TritonX-100 was placed in the −80 °C refrigerator for half an hour to release the tyrosinase in B16F10 cells; 80 μL 1 mg/mL LOPA was added and incubated at 37 °C for 1 h. Then it was centrifuged, the supernatant was taken, and its absorbance was measured at 492 nm.

### 2.8. Statistical Analysis

All experiments were repeated three times. All of the data were analyzed by e GraphPad Prism 5.0 statistical software and expressed as means ± standard deviation (SD). Data analysis used unpaired *t* test to test whether there was a significance between the two groups of data. *p* < 0.05 was considered statistically significant.

## 3. Results

### 3.1. Virtual Screening

The CB1 crystal structure (PDB No.: 5TGZ) was downloaded from the PDB database. The active binding site was determined, according to the position of the coordinates of the known ligands in the target protein [42]. Docking CB1 with CBD yielded an excellent score, and the results are shown in Table 1 and Table 2. From Table 2, it can be seen that cannabidiol formed a hydrogen bonding with hydroxyl group of residue SER383, and the benzene ring formed Pi–Pi stacking with residue PHE170. Therefore, the skeleton, shown in Figure 1, was submitted to InterBioScreen and Specs databases to search for compounds with 70% similarity with CBD. As a result, 422 compounds remained.

As shown in Figure 1, the CDOCK module was used to dock the remaining compounds with CB1, and 357 compounds with a better score than CBD were left. Then, Lipinski’s rule of five and Veber’s rules were further applied to remove the compounds that were weak drug likenesses., which resulted in 228 compounds remaining for the following process. Then, the Glide XP module with higher docking precision was implemented, and 153 compounds with a docking score less than −7 were obtained. Via the hierarchical clustering, these compounds were clustered into 10 groups. Within these clusters, four compounds (S-88614, S-88745, S-92151, S-92153) owning similar binding modes with CBD, as shown in Table 1, were chosen for the subsequent experiments, because the nitrogen and oxygen atoms of these compounds can form one or two hydrogen bonds with the key residues GLN116 or HIS181, and benzene ring to form Pi–Pi interactions with the key residues PHE102, PHE170 or HIS178 (Table 2). Their rotatable bonds are less than or equal to 10, and their polar surface area is equal to or <140 Å^2^, indicating that these compounds have a good bioavailability [43]. Furthermore, cannabidiol shows antioxidant properties because hydroxyl is easy to be oxidized [44], we can deduce that the four compounds may also have antioxidant performance due to the functional group of phenolic hydroxyl. The hit compounds are listed in Table 1.

### 3.2. Extracellular Antioxidant Test

**Figure 2:**
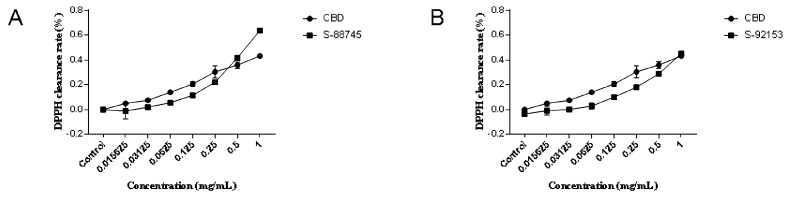
The graph of DPPH scavenging ability. (**A**) The DPPH scavenging abilities of CBD and its skeleton derivative, S-88745. (**B**) The DPPH scavenging ability of CBD and its skeleton derivative, S-92153. The control group was not treated with CBD, S-88745 and S-92153, and only contained DPPH solution. The experiments were repeated three times. Each bar illustrates the average ± standard deviation (SD) counted from three experiments.

**Figure 3:**
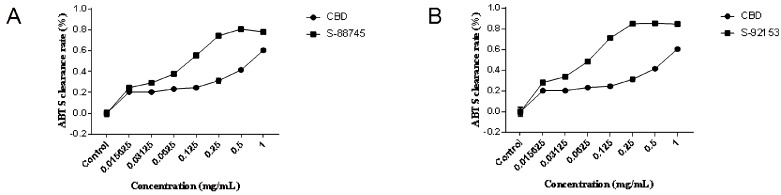
The graph of ABTS scavenging ability. (**A**) The ABTS scavenging abilities of CBD and its skeleton derivative, S-88745. (**B**) The ABTS scavenging ability of CBD and its skeleton derivative, S-92153. The control group was not treated with CBD, S-88745 and S-92153, and only contained ABTS solution. The experiments were repeated three times. Each bar illustrates the average ± standard deviation (SD) counted from three experiments.

The ABTS and DPPH tests were used, as stated in Section 2.3, to assess the molecular antioxidant abilities. The results indicated that compounds S-88745 and S-92153 had a stronger antioxidant capacity than cannabidiol, as shown in Figure 2 and Figure 3, while S-88614 and S-92151 were weaker (data not shown). In the DPPH scavenging experiment, the DPPH scavenging rate of S-88745 was significantly higher than that of CBD when the concentration was higher than 0.5 mg/mL (Figure 2A), and the ability of S-92153 to scavenge DPPH free radicals was similar to CBD (Figure 2B). In the ABTS scavenging experiment, the antioxidant capacities of S-88745 and S-92153 were significantly stronger than that of CBD. The maximum scavenging rate of S-88745 and S-92153 can reach 80% and 85%, respectively (Figure 3A,B). Therefore, it is worthy to further test the anti-inflammatory, anti-wrinkle and whitening effects of S-88745 and S-92153.

### 3.3. Anti-Inflammatory Activity Test

#### 3.3.1. Cytotoxicities Measurement

**Figure 4:**
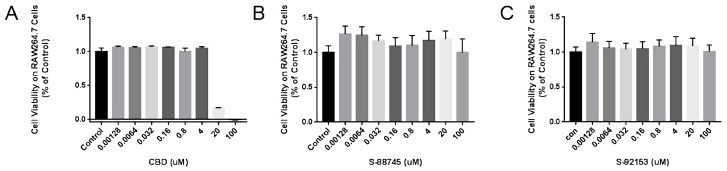
Cytotoxicity of (**A**) CBD, (**B**) S-88745 and (**C**) S-92153, on RAW264.7 cells. RAW264.7 cells were treated with cannabidiol, S-88745 or S-92153 for 24 h, and MTT assay was carried out to detect the cell viability. The control group was not treated with CBD, S-88745 and S-92153. The experiments were repeated three times. Each bar illustrates the average ± standard deviation (SD) counted from three experiments.

The cytotoxicity of CBD, S-88745 and S-92153 (0.000256–100 μM) on RAW264.7 cells was measured by MTT assay. The results are shown in Figure 4, from where we can see that CBD showed a significant cytotoxicity effect on RAW264.7 cells in the range of 20–100 μM (Figure 4A), while S-88745 and S-92153 did not show any cytotoxicity effect on RAW264.7 cells (Figure 4B,C). In order to study the anti-inflammatory effect of S-88745 and S-92153, the levels of NO, IL-6, iNOS and COX expression were determined in RAW264.7 cells at non-cytotoxic concentrations (determined as 0.04, 0.4 and 4 μM).

#### 3.3.2. Suppression of NO

**Figure 5:**
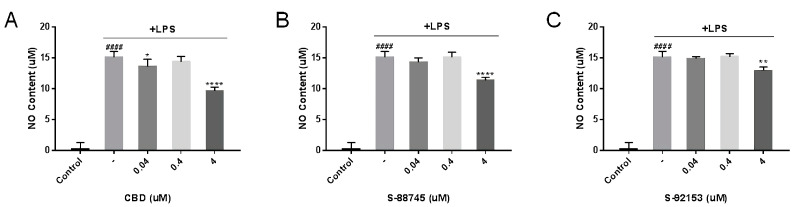
Inhibition of (**A**) CBD, (**B**) S-88745 and (**C**) S-92153 on LPS-induced nitric oxide (NO) in RAW 264.7 macrophages. The control group was not stimulated by LPS. The LPS-treated group (-) was stimulated by LPS to develop inflammation. The experiments were repeated three times. Each bar illustrates the average ± standard deviation (SD) counted from three experiments. *p* < 0.05 was considered statistically significant. *p* < 0.05 was considered statistically significant. * *p* < 0.05 compared to the LPS−treated group; ** *p* < 0.01 compared to the LPS−treated group; **** *p* < 0.0001 compared to the LPS−treated group; #### *p* < 0.0001 compared to the control group.

NO, an indicator of the inflammatory degree, is synthesized by inducible nitric oxide synthase (iNOS) from L-arginine. NO serves an important pathological role. Lipopolysaccharide (LPS) stimulates inflammatory stress, inducing a large number of NO [37]. As shown in Figure 5, RAW264.7 cells treated by LPS showed a sharp increase in NO production, but CBD, S-88745 and S-92153 significantly reduced the NO secretion to 4 μM (36%, 24.7%, 14.5%).

#### 3.3.3. Inhibitory Effect on IL-6 Production

**Figure 6:**
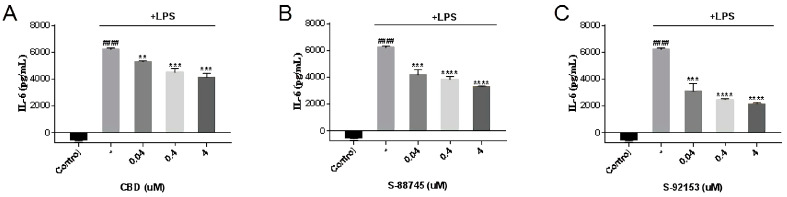
Effects of (**A**) CBD, (**B**) S-88745 and (**C**) S-92153 on IL-6 induced by LPS. In addition to an untreated control group and the LPS−treated group, RAW264.7 cells pretreated with CBD, S-88745 and S-92153 at 0.04, 0.4, and 4 µM for 1.5 h, and then treated all groups with LPS, except the control group for 20 h. The levels of IL-6 in the culture supernatants of RAW264.7 macrophages were then detected by ELISA. The experiments were repeated three times. Each bar illustrates the average ± standard deviation (SD) counted from three experiments. *p* < 0.05 was considered significant. *p* < 0.05 was considered statistically significant. ** *p* < 0.01 compared to the LPS−treated group; *** *p* < 0.001 compared to the LPS−treated group; **** *p* < 0.0001 compared to the LPS−treated group; #### *p* < 0.0001 compared to the control group.

As can be seen from Figure 6, IL-6 significantly increased after LPS was added to stimulate inflammation. CBD and the two compounds can counteract the effect in a concentration dependent manner, as reported [45,46]. CBD (4 μM) reduced the release of IL-6 induced by LPS (Figure 6A) by 33.4%, S-88745 (4 μM) decreases by 47% (Figure 6B) and S-92153 (4 μM) decreases by 65% (Figure 6C), compared with the LPS group. Comparing with CBD, the hit compounds S-88745 and S-92153 can decline IL-6 more efficiently, indicating their better anti-inflammatory effects.

#### 3.3.4. Effects on Protein Expression of iNOS and COX-2

**Figure 7:**
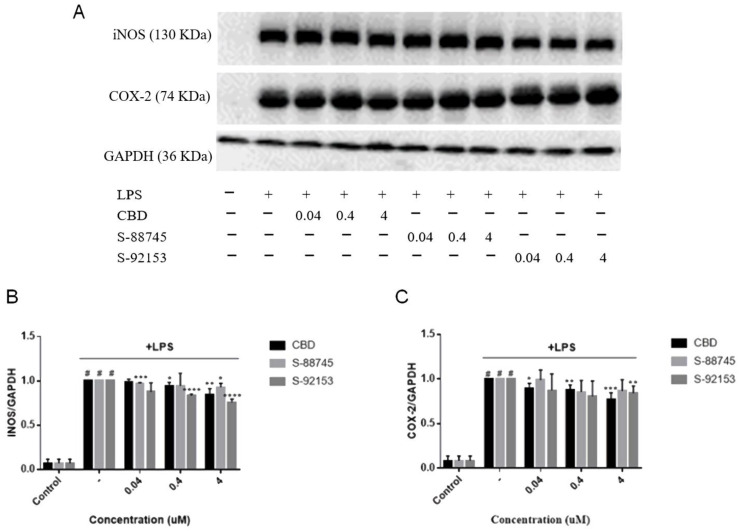
Effects of CBD, S-88745 and S-92153 on the expression of iNOS and COX-2 induced by LPS. In addition to an untreated control group and the LPS−treated group, RAW264.7 cells pretreated with CBD, S-88745 and S-92153 at 0.04, 0.4, and 4 µM for 1.5 h, and then treated all groups with LPS, except the control group for 20 h. The levels of iNOS and COX-2 in the culture supernatants of RAW264.7 macrophages were then detected by western blot. (**A**) iNOS and COX-2 protein expression levels. (**B**) Relative ration analysis of iNOS expression. (**C**) Relative ration analysis of COX-2 expression. The experiments were repeated three times. Each bar illustrates the average ± standard deviation (SD) counted from three experiments. *p* < 0.05 was considered statistically significant. * *p* < 0.05 compared to the LPS−treated group; ** *p* < 0.01 compared to the LPS−treated group; *** *p* < 0.001 compared to the LPS−treated group; **** *p* < 0.0001 compared to the LPS−treated group; # *p* < 0.0001 compared to the control group.

In order to evaluate the effects of CBD and its derivatives on proinflammatory mediators, the protein expressions of iNOS and COX-2 were determined. CBD, S-88745 and S-92153 can decrease the expression of iNOS protein, especially S-92153, which has the most significant effect, decreased the expression of iNOS by 11%, compared to the LPS-treated model group. Because NO is synthesized by iNOS from L-arginine, we can deduce that S-88745 and S-92153 maybe reduce the NO content by inhibiting the expression of iNOS (Figure 5 and Figure 7C).

The results are shown in Figure 4. It can be seen that CBD (4 μM) decreased the expression of COX-2 by 22.9% in a concentration dependent manner. S-92153 (4 μM) decreased the expression of COX-2 by 13.4% also in a concentration dependent manner. Though S-88745 reduced the expression of COX-2 to some extent, there was no significant difference, compared to the LPS-treated model group.

### 3.4. Anti-Wrinkle Activity Test

#### 3.4.1. Cytotoxicity Measurement on HFF-1 Cell Line

**Figure 8:**
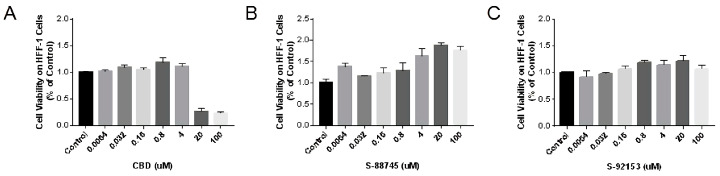
Cytotoxicity of (**A**) CBD and its derivatives, (**B**) S-88745 and (**C**) S-92153, on HFF-1 cells. HFF-1 cells were treated with cannabidiol, S-88745 or S-92153 for 72 h, and MTT assay was carried to detect the cell viability. The experiments were repeated three times. Each bar illustrates the average ± standard deviation (SD) counted from three experiments.

Inhibiting the proliferation of fibroblasts will lead to the degradation of collagen, which is one of the reasons for wrinkles. Therefore, the MTT method was used to detect the effects of compounds on the proliferation of HFF-1. The results are shown in Figure 8, in which CBD showed a significant cytotoxicity effect on HFF-1 cells in the range of 20–100 μM (Figure 8A). On the contrary, S-88745 and S-92153 promoted fibroblast proliferation in the range of 20–100 μM. These two compounds markedly promoted the proliferation of fibroblasts at 20 μM. The survival rate of S-88745 in 20 μM cells was 188.3%, compared to the control group (Figure 8B), and that of S-92153 in 20 μM cells was 121.7% (Figure 8C). Therefore, S-88745 and S-92153 may increase the content of extracellular matrix by promoting fibroblast proliferation (Figure 8B,C).

#### 3.4.2. Fibroblast Damage Repairment Evaluation

**Figure 9:**
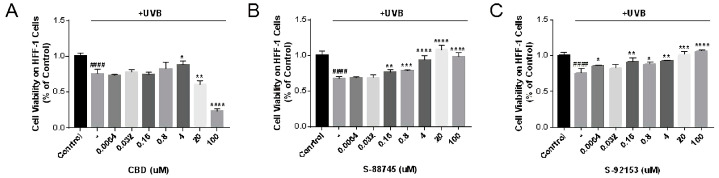
Cell viability of (**A**) CBD and its derivatives, (**B**) S-88745 and (**C**) S-92153, on HFF-1 cells. HFF-1 cells were treated with cannabidiol, S-88745 or S-92153 for 4 h, then UV intensity of 70 mW/cm was irradiated for 5 min. Following another 20 h, MTT assay was carried out to detect the cell viability. Each bar illustrates the average ± standard deviation (SD) counted from three experiments. The experiments were repeated three times. Each bar illustrates the average ± standard deviation (SD) counted from three experiments. *p* < 0.05 was considered significant. *p* < 0.05 was considered statistically significant.* *p* < 0.05 compared to the UV−treated group; ** *p* < 0.01 compared to the UV−treated group; *** *p* < 0.001 compared to the UV−treated group; **** *p* < 0.0001 compared to the UV−treated group; #### *p* < 0.0001 compared to the control group.

Ultraviolet rays entering the atmosphere are mainly UVA (315–400 nm) and UVB (280–315 nm) [11,13,23]. UVB is more genotoxic and causes more sunburn. The oxidative stress in skin induced by UVB radiation generates reactive oxygen species, such as hydrogen peroxide, superoxide anion and hydroxyl radical, which leads to biological reactions in the skin, such as inflammation induction, melanin production, photoaging fibroblast death, etc. [23]. We chose UVB to build the UV irradiation injury model. As shown in Figure 9A, CBD can promote the proliferation of HFF-1 cells in the concentration of 4 μM, but it accelerated the damage to the fibroblasts in the range of 20–100 μM. Therefore, the range of CBD concentrations that can be used to treat the light injury is relatively narrow. On the contrary, S-88745 and S-92153 can significantly repair the damage caused by ultraviolet radiation even at high concentrations. The survival rate of HFF-1 cells treated with S-88745 (4–100 μM) recovered to the level before UV damage (Figure 9B). S-92153 also has an excellent ability to repair damage caused by UV radiation in 20–100 μM (Figure 9C). Considering the anti-ultraviolet effect of them, 0.16–4 μM was selected for the following experiments.

#### 3.4.3. Intracellular Antioxidant Activity

**Figure 10:**
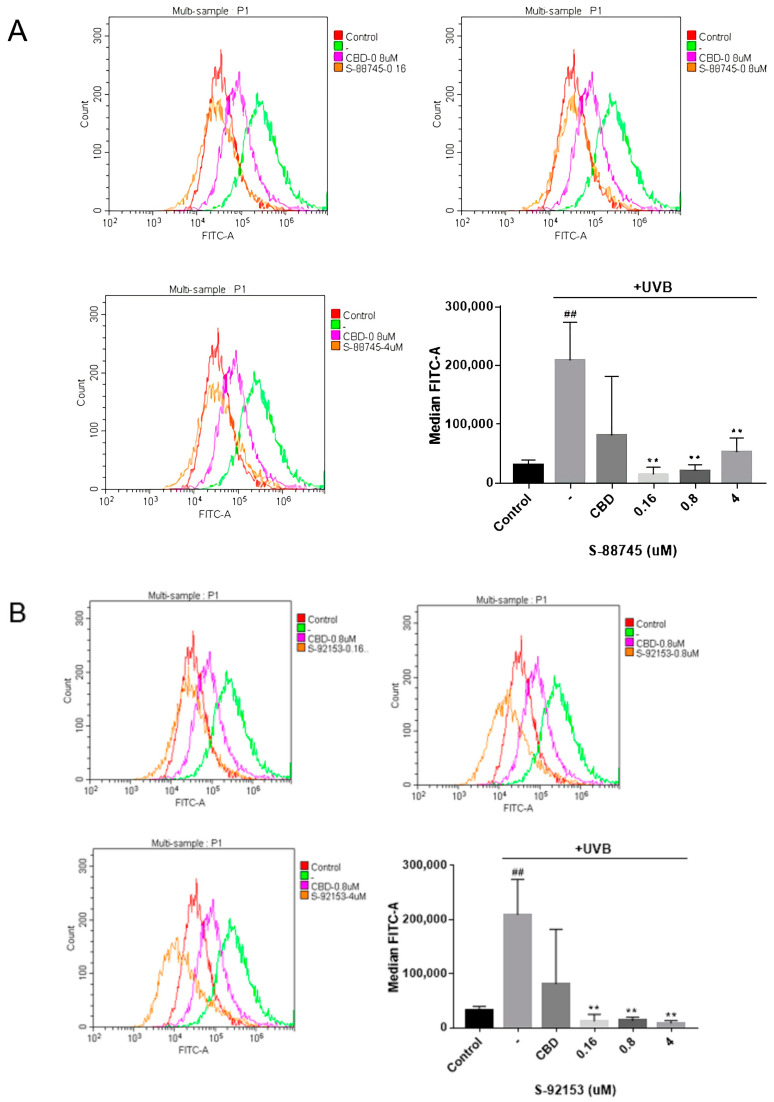
Inhibition of CBD, S-88745 and S-92153 on UV-induced ROS in HFF-1. Each bar illustrates the average ± standard deviation (SD) counted from three experiments. (**A**) ROS content of S-88745 and CBD (0.8 μM). (**B**) ROS content of S-92153 and CBD (0.8 μM). The control group was not stimulated by UV. The UV-treated group (-) produced a lot of reactive oxygen species after UV stimulation. The experiments were repeated three times. Each bar illustrates the average ± standard deviation (SD) counted from three experiments. *p* < 0.05 was considered statistically significant. ** *p* < 0.01 compared to the UV−treated group; ## *p* < 0.01 compared to the control group.

ROS is related to many pathological states of the skin, such as a serious loss of extracellular matrix leading to facial wrinkles [5,18,47]. Therefore, we carried out the intracellular antioxidant experiments to evaluate the screened compounds. As shown in Figure 10A, UV radiation caused the increase in intracellular ROS levels in HFF-1, compared to control group. The clearance rate of CBD reactive oxygen species was 61.2% at 0.8 μM, which was consistent with the literature [36]. The intracellular antioxidant capacity of S-88745 is better than that of CBD, and the clearance rate of active oxygen is 89.8% at 0.8 μM. Furthermore, the level of active oxygen has returned to the level before UV damage (Figure 10A). Compound S-92153 has the best scavenging capacity of intracellular ROS, and the scavenging rate of ROS is 93.6% at 0.8 μM, which indicates that S-92153 has an excellent antioxidant capacity (Figure 10B).

#### 3.4.4. Fibroblast Apoptosis Experiment

**Figure 11:**
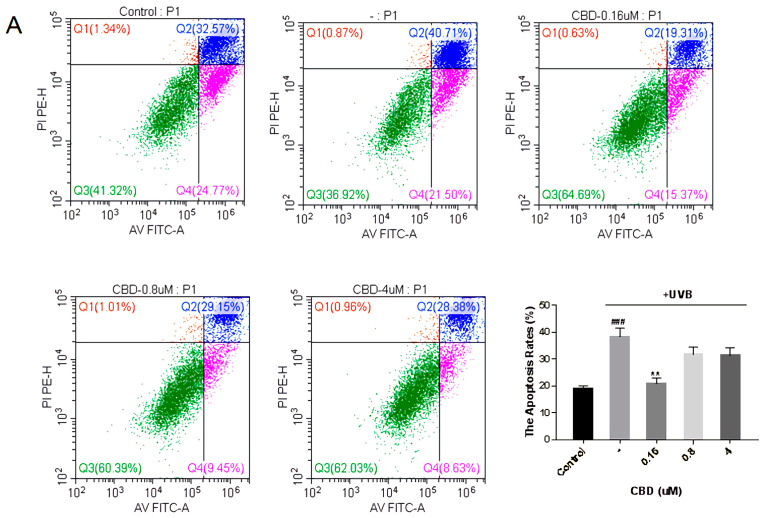

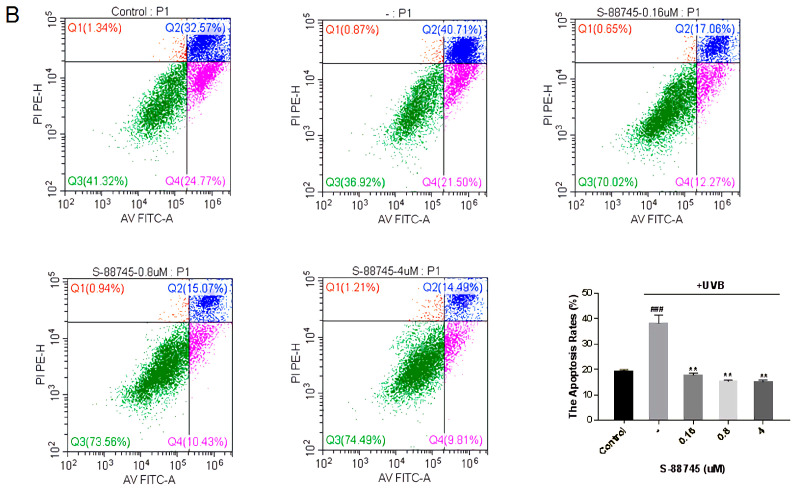

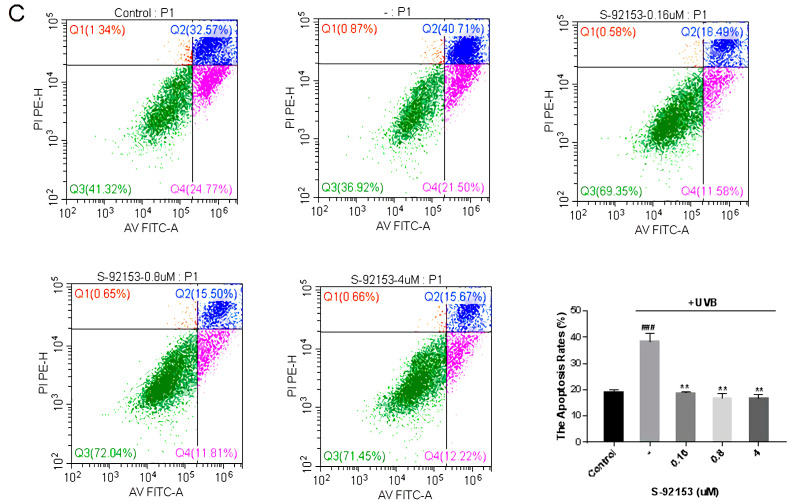
Inhibition of (**A**) CBD, (**B**) S-88745 and (**C**) S-92153 on UV-induced apoptosis in HFF-1. The control group was not stimulated by UV. The UV-treated group (-) produced a lot of reactive oxygen species after UV stimulation. The experiments were repeated three times. Each bar illustrates the average ± standard deviation (SD) counted from three experiments. *p* < 0.05 was considered statistically significant. ** *p* < 0.01 compared to the UV−treated group; ### *p* < 0.001 compared to the control group.

Oxidative stress caused by ultraviolet rays produces a large amount of oxygen, which will break the function of DNA and promote cell apoptosis. It leads to a decrease in the number of fibroblasts in the dermis, and finally leads to a decrease in the content of extracellular matrix, resulting in wrinkles. Annexin V-FITC/PI staining was used to detect the effect of the compounds on the proliferation of fibroblasts, and flow cytometry was used to analyze Annexin V-FITC/PI staining. As shown in Figure 11, the apoptosis rate was increased to 40.71% after UV irradiation. CBD can significantly alleviate cell apoptosis caused by UV, with the best effect at 0.16 μM. Compared with the UV group, the apoptosis rate decreased by 53% (Figure 11A). S-88745 and S-92153 can also significantly inhibit cell apoptosis, and the effect of inhibiting cell apoptosis becomes more obvious with the increase of concentration. Compared with the ultraviolet group, the apoptosis rate decreases by 64% and 61.5% at 4 μM, respectively, for S-88745 and S-92153 (Figure 11B,C).

#### 3.4.5. Effects Evaluation on Collagen, Elastin and Fibronectin Production

Collagen, which is responsible for the strength and resilience of skin, provides support for the epidermal structure. Its degradation can cause skin laxity. Elastin fibers maintain stretch conditions and provide recoil to the tissues. Fibronectin is widely involved in cell migration, adhesion and tissue repair. We detected the effects of compounds on the intracellular collagen, elastin and fibronectin content in fibroblasts.

**Figure 12:**
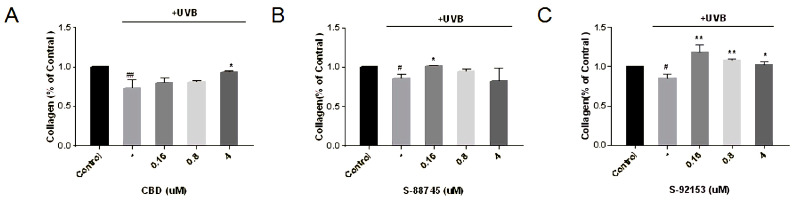
Production of (**A**) CBD, (**B**) S-88745 and (**C**) S-92153 on UV-stimulated collagen in HFF-1. The control group was not stimulated by UV. The UV-treated group (-) was stimulated by UV to cause ECM degradation. The experiments were repeated three times. Each bar illustrates the average ± standard deviation (SD) counted from three experiments. *p* < 0.05 was considered statistically significant. * *p* < 0.05 compared to the UV−treated group; ** *p* < 0.01 compared to the UV−treated group; # *p* < 0.05 compared to the control group; ## *p* < 0.01 compared to the control group.

The results of collagen were shown in Figure 12, from where it can be seen that after UV irradiation, the content of collagen decreased by 34%, compared to only the UVB group. CBD works only at a high concentration of 4 μM, and the collagen content has increased by 27.7%, which is near the level before UV damage (Figure 12A). S-88745 and S-92153 work at low concentrations, and the collagen content at 0.16 μM increased by 19% and 40.8%, respectively (Figure 12B,C).

**Figure 13:**
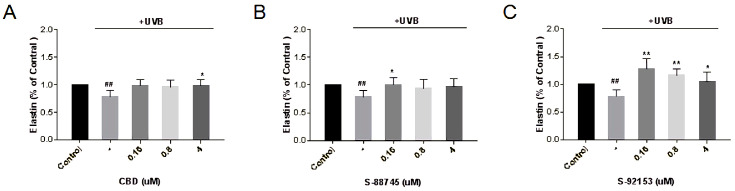
Production of (**A**) CBD, (**B**) S-88745 and (**C**) S-92153 on UV-stimulated elastin in HFF-1. The control group was not stimulated by UV. The UV-treated group (-) was stimulated by UV to cause ECM degradation. The experiments were repeated three times. Each bar illustrates the average ± standard deviation (SD) counted from three experiments. *p* < 0.05 was considered statistically significant. * *p* < 0.05 compared to the UV−treated group; ** *p* < 0.01 compared to the UV−treated group; ## *p* < 0.01 compared to the control group.

As shown in Figure 13, after ultraviolet radiation, the elastin content in fibroblasts decreased by 22%. It is obvious that CBD, S-92153 and S-92153 can alleviate the loss of elastin caused by UV irradiation, and even restore the skin to the level before UV injury. However, the abilities of S-88745 and S-92153 to improve collagen are negatively correlated to the concentration, and the effect is best at 0.16 μM (Figure 13B,C). As a result, CBD, S-88745 and S-92153 can increase the elastin by 38.8%, 17.9% and 63% at 0.16 μM, compared to the model group, respectively (Figure 13).

**Figure 14:**
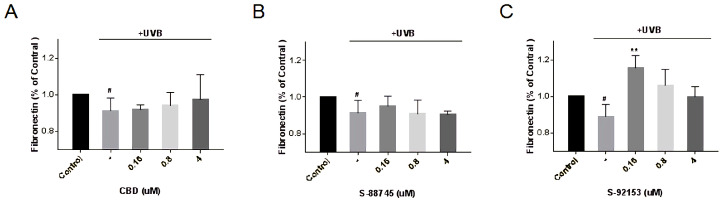
Production of (**A**) CBD, (**B**) S-88745 and (**C**) S-92153 on UV-stimulated fibronectin in HFF-1. The control group was not stimulated by UV. The UV-treated group (-) was stimulated by UV to cause ECM degradation. The experiments were repeated three times. Each bar illustrates the average ± standard deviation (SD) counted from three experiments. *p* < 0.05 was considered statistically significant. ** *p* < 0.01 compared to the UV−treated group; # *p* < 0.05 compared to the control group.

The results of fibronectin were shown in Figure 14. It is obvious that UV can promote the decomposition of fibronectin, and the content of fibronectin decreases by 9%, compared to the control group. Unfortunately, CBD and S-88745 did not significantly promote fibronectin production. Only S-92153 could promote fibronectin production and the effect was best at 0.16 μM, which increased the fibronectin content by 26.7%.

Based on the above results, S-92153 is expected to rapidly restore extracellular matrix content, such as collagen, elastin and fibrin in a short time when dealing with UV irradiation damage.

### 3.5. Whitening Activity Test

#### 3.5.1. Cytotoxicity Measurements on B16F10

**Figure 15:**
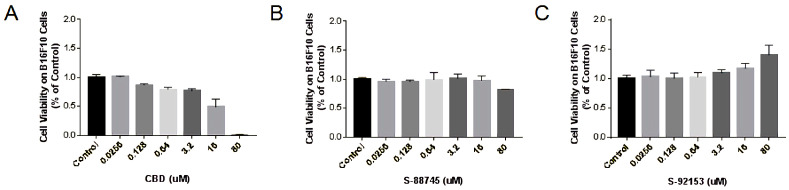
Cytotoxicity of (**A**) CBD and its derivatives, (**B**) S-88745 and (**C**) S-92153, on B16F10 cells. B16F10 cells were treated with cannabidiol, S-88745 or S-92153 for 72 h, and MTT assay was carried to detect the cell viability. The control group was not treated with CBD, S-88745 and S-92153. The experiments were repeated three times. Each bar illustrates the average ± standard deviation (SD) counted from three experiments.

Following the exploration of the anti-inflammatory and anti-wrinkle effects, we continued to study the whitening effects of CBD and its derivatives. At first, the cytotoxicities on the proliferation of B16F10 melanoma cells were studied by using MTT. The results are shown in Figure 15, from where we can see that CBD can significantly inhibit the activity of B16F10 cells in a concentration-dependent manner (Figure 15A). Compound S-88745 demonstrates cytotoxicity only at 80 μM (Figure 15B), while S-92153 was not cytotoxic to B16F10 cells (Figure 15C). Based on the MTT test results, 0.0256–0.64 μM was selected for subsequent experiments.

#### 3.5.2. Whitening Activity Tests

**Figure 16:**
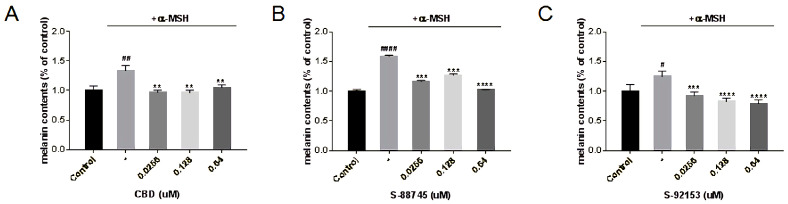
Inhibition of (**A**) CBD, (**B**) S-88745 and (**C**) S-92153 on α-MSH-induced melanogenesis in B16F10. The control group was not stimulated by α-MSH. The α-MSH -treated group (-) was stimulated by α-MSH to produce pigmentation. The experiments were repeated three times. Each bar illustrates the average ± standard deviation (SD) counted from three experiments. *p* < 0.05 was considered statistically significant. ** *p* < 0.01 compared to the α-MSH −treated group; *** *p* < 0.001 compared to the α-MSH −treated group; **** *p* < 0.0001 compared to the α-MSH−treated group; # *p* < 0.05 compared to the control group; ## *p* < 0.01 compared to the control group; #### *p* < 0.0001 compared to the control group.

In order to detect the whitening effects of CBD, S-88745 and S-92153, α-MSH was used to stimulate B16F10 melanoma cells to secrete melanin. Then compounds were added with different concentrations, and the sodium hydroxide lysis method was used to detect the intracellular melanin content. The results are shown in Figure 16. It can be seen that all three compounds can reduce the intracellular melanin content to some extent, among which S-92153 significantly reduced the content of melanin in a concentration dependent manner (Figure 16C).

#### 3.5.3. The Effects on Tyrosinase Activity

**Figure 17:**
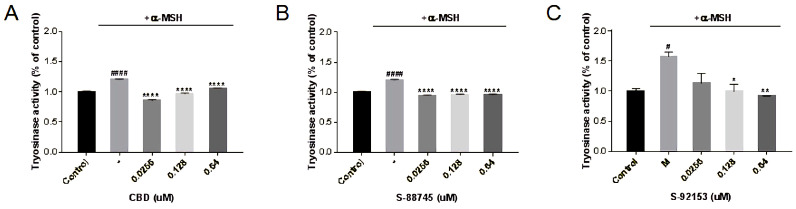
Inhibition of (**A**) CBD, (**B**) S-88745 and (**C**) S-92153 on α-MSH-induced tyrosinase activity in B16F10. The control group was not stimulated by α-MSH. The α-MSH -treated group (-) was stimulated by α-MSH to produce pigmentation. The experiments were repeated three times. Each bar illustrates the average ± standard deviation (SD) counted from three experiments. *p* < 0.05 was considered statistically significant. * *p* < 0.05 compared to the α-MSH −treated group; ** *p* < 0.01 compared to the α-MSH −treated group; **** *p* < 0.0001 compared to the α-MSH−treated group; # *p* < 0.05 compared to the control group; #### *p* < 0.0001 compared to the control group.

Tyrosinase plays an important role in the formation of melanin, so we investigated whether CBD and its derivatives can affect the activity of tyrosinase. As shown in Figure 17, the ability of CBD to inhibit tyrosinase was negatively correlated with the concentration (Figure 17A). At 0.16 μM, the activity of tyrosinase was 70.9%, of the group with only added melanin. The ability of CBD to inhibit melanin is also negatively related to the concentration, because CBD may reduce the production of melanin by inhibiting the activity of tyrosinase. S-88745 significantly decreased tyrosinase activity at 0.0256–0.64 μM (Figure 17B). S-92153 decreased the activity of tyrosinase in a concentration dependent manner. At 0.64 μM, the activity of tyrosinase was only 58.8% of the model group. The ability of S-92153 to inhibit melanin is also negatively related to the concentration, because S-92153 may inhibit melanin production by inhibiting the activity of tyrosinase (Figure 17C).

## 4. Discussion

Based on the experimental results, we further explore this study. Cannabidiol is one of the few cosmetic ingredients with multiple skincare benefits, but its usage is limited due to the limited resources and rules acting in many countries. Therefore, we intend to find substitutes to CBD that have similar skincare benefits by combining computer-aided drug designs and experimentation methods.

We studied the action mode between CBD and target protein CB1 to find the molecular key skeleton, and virtually screened huge natural products databases to search for compounds with a 70% structural similarity to the skeleton. Following Lipinski’s rule of five, Veber’s rules screening, and docking screening, 153 compounds with a docking score of less than −7 and drug-like properties were retained. Via the hierarchical clustering, these compounds were clustered into 10 groups. Within these clusters, four compounds (S-88614, S-88745, S-92151, S-92153) were chosen and purchased for the subsequent experiments. These four compounds have phenolic hydroxyl structures similar to CBD, which may be the basis of their antioxidant properties.

Then, the determination of extracellular tests (ABTS and DPPH) and intracellular reactive oxygen species was used to verify the antioxidant properties of the compounds, and S-88745 and S-92153 showed great potential.

Experimental results show that cannabidiol and its derivatives can reduce the production of NO, IL-6, iNOS and COX-2, thereby producing anti-inflammatory effects. Our research shows that the tested compounds can reduce NO and iNOS expression, and they may down regulate the content of NO by inhibiting the expression of iNOS. They also can compensate for the UV damage. The experimental results indicated that these compounds can increase the content of collagen, elastin and fibronectin, and can affect the apoptosis of fibroblasts. IL-6 and COX-2 can upregulate the expression of MMPs, thus promoting the degradation of extracellular matrix. Cannabinol and its derivatives can reduce the degradation of collagen, elastin and fibronectin caused by ultraviolet radiation, which may be related to their ability to down regulate the expression of IL-6 and COX-2. In addition, ultraviolet radiation can cause fibroblast death, thereby reducing the overall expression of the extracellular matrix. Compared with the model group, the survival rate of cells treated with CBD and its derivatives was greatly improved, and the apoptosis rate was also decreased. Therefore, cannabinol and its derivatives can alleviate the collagen caused by ultraviolet radiation. The degradation of elastin and fibronectin may be related to their increased cell survival rate and decreased apoptosis rate after ultraviolet radiation. All of these results indicated that cannabidiol and its derivatives have anti-wrinkle effects. Furthermore, they can significantly reduce the activity of tyrosinase and adjust the generation of melanin. It is worth mentioning that the anti-inflammatory and anti-wrinkle whitening effect of S-88745 and S-92153 are stronger than that of CBD, which is consistent with the antioxidant effect. Therefore, it can be inferred that inflammation, loss of ECM, and melanin precipitation may be affected by reactive oxygen species by the common MAPK pathway.

The abundant generation of reactive oxygen species activates the MAPK pathway, which then excites NF-κB, MMPs and MITF. NF-κB can activate downstream inflammatory factors and inflammation-related proteins. MMPs promote ECM degradation, leading to skin laxity and loss of elasticity. MITF activates tyrosinase and other enzymes involved in melanin synthesis. It can be seen that MAPK channel is related to a variety of skin problems. Studies suggested that CBD modules the activation of the MAPK path through JNK and ERK signaling path, and then activate NF-κB Channel [48,49]. In addition, CBD can significantly reduce the expression level of p38, ERK, JNK mRNA and the expression of MMPs [50]. At present, the mechanism of reducing melanin content in CBD is not clear. This study indicated that cannabidiol may have anti-inflammatory, anti-wrinkle and whitening effects by inhibiting the MAPK pathway. CBD and its derivatives generate multiple skin care effects through the MAPK pathway; therefore drug design can be carried out for this pathway.

## 5. Conclusions

In this study, the structure of CBD was optimized to obtain two compounds with similar multifunctional skin care efficacies as CBD. Firstly, The action mode between CBD and target protein CB1 was analyzed, and the action skeleton was put into the natural products database. Following the molecular docking, drug likeness evaluation and cluster analysis, four compounds with strong binding abilities to CB1 were obtained. ABTS and DPPH radical scavenging experiments showed that S-88745 and S-92153 had a stronger antioxidant capacity than CBD compounds. MTT, ELISA, western blot, flow cytometry and other experimental methods were used to analyze the anti-inflammatory, anti-wrinkle and whitening effects of these two compounds. These results proved that the two compounds S-88745 and S-92153 are superior to CBD, in terms of antioxidant, anti-wrinkle and whitening efficacy with a lower cytotoxicity. These two compounds can replace cannabinol in the cosmetics industry, which can solve the problem of insufficient sources and restricted usage of CBD. This may be a new solution to skin problems.

## Figures and Tables

**Table 1:** The molecular structures and docking scores of CBD and the selected compounds, by virtual screening.

No.	Database	Structure	Docking Score
CBD	InterBioScreen	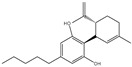	−10.274
S-88614	InterBioScreen	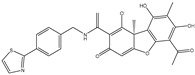	−8.494
S-88745	InterBioScreen	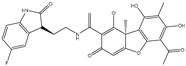	−9.267
S-92151	InterBioScreen	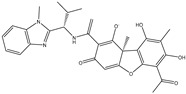	−7.285
S-92153	InterBioScreen	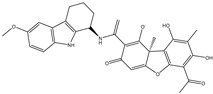	−8.219

**Table 2:** The stereo view and floor plan view of the docking results of CBD and the selected compounds.

No.	Stereo View of the Docking Results	Floor Plan View of the Docking Results
CBD	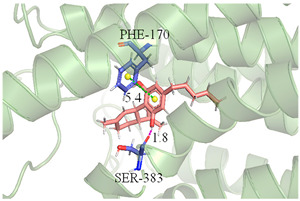	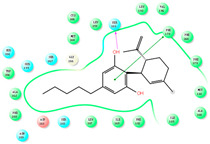
S-88614	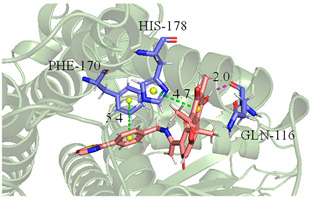	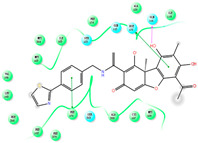
S-88745	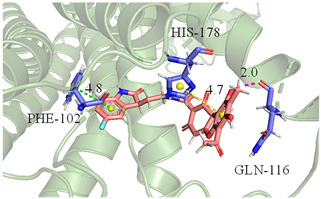	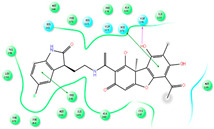
S-92151	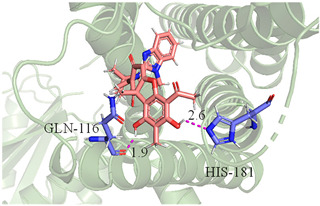	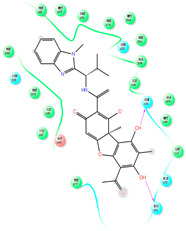
S-92153	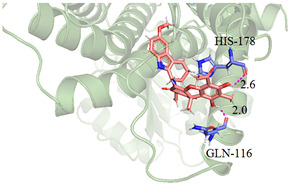	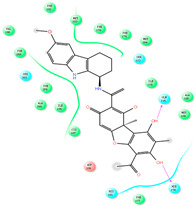

## Data Availability

The data presented in this study are available in Supplementary Material.

## Supplementary Materials

The following supporting information can be downloaded at: https://www.mdpi.com/article/10.3390/antiox12020314/s1.
